# Characterization of various tandem solar cells: Protocols, issues, and precautions

**DOI:** 10.1002/EXP.20220029

**Published:** 2023-04-10

**Authors:** Jae Hyun Park, Sun Kyung Hwang, Su Geun Ji, Jin Young Kim

**Affiliations:** ^1^ Department of Materials Science and Engineering Seoul National University Seoul Republic of Korea; ^2^ Research Institute of Advanced Materials Seoul National University Seoul Republic of Korea

**Keywords:** characterizations, four‐terminals, tandem solar cells, three‐terminals, two‐terminals

## Abstract

In the search for a more efficient solar cell, various types of tandem solar cells (TSCs) have been actively developed worldwide as the performances of the single junction solar cells approach their theoretical limits. Meanwhile, various materials and structures are adopted in TSCs, which makes their characterizations and comparison difficult. Along with the classical monolithic TSC, which exhibits two electrical contacts, devices with three or four electrical contacts have been widely studied as a more performing alternative of commercialized solar cells. For a fair and accurate evaluation of the device performance of TSCs, understanding the effectiveness and limitations of the characterization of the different types of TSCs is crucial. In this paper, we summarize various types of TSCs and discuss their characterization methods.

## Graphical abstract

Various structures are adopted in tandem solar cells (TSCs) to overcome the efficiency limit of the single junction solar cell. Fair and accurate evaluation of the various TSCs requires understanding the effectiveness and limitations of their characterization methods. Various types of TSCs are summarized. And characterization methods of TSCs are discussed.

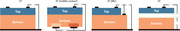

## 1 INTRODUCTION

Single‐junction solar cells (SJSCs) consisting of a single absorber material have a fundamental (detailed balance) limit of efficiency, where more than 65% of the solar energy is lost due to the optical and thermalization losses. However, tandem solar cells (TSCs) consisting of two or more absorber materials with different bandgap values utilize the solar spectrum more effectively by reducing the thermalization losses, leading to a higher power conversion efficiency than the SJSCs. Recently, emerging TSCs consisting of two or more economically viable solar cells are attracting strong interest as their certified efficiency (31.3% for the perovskite(PVSK)/Si tandem) has surpassed the highest efficiency of SJSCs (29.1% for GaAs solar cells).^[^ ^1^ ^]^ However, unlike conventional multijunction solar cells based on III‐V materials, the emerging tandems can have a broader selection of material combinations and device configurations. In addition to existing problems such as spectral mismatch, this can lead to new complications in performance measurement. In particular, the number of electrical contacts can vary from two to four, and the subcells can be electrically connected in series or in parallel, all of which require their own measurement protocols with different levels of accuracy. TSCs with three or four contacts often benefit from a higher degree of freedom during measurement, mostly owing to the presence of the additional electrodes, than the two‐terminal (2T) counterpart whose characterization is limited by the series connection of the subcells. A fair and accurate evaluation of the device performance and successful commercialization of TSCs requires crucial understanding of the effectiveness and limitations of the specific measurement protocols for the different types of TSCs. While TSCs with two contacts have standard measurement methods based on the existing standards for single‐junction devices,^[^ ^2^ ^]^ no standard method is available for TSCs with more than two electrical contacts. In this review article, we summarize commonly used measurement protocols for various types of TSCs and provide several insights into their limitations.

## 2 CLASSIFICATION OF TSCs

**FIGURE 1:**
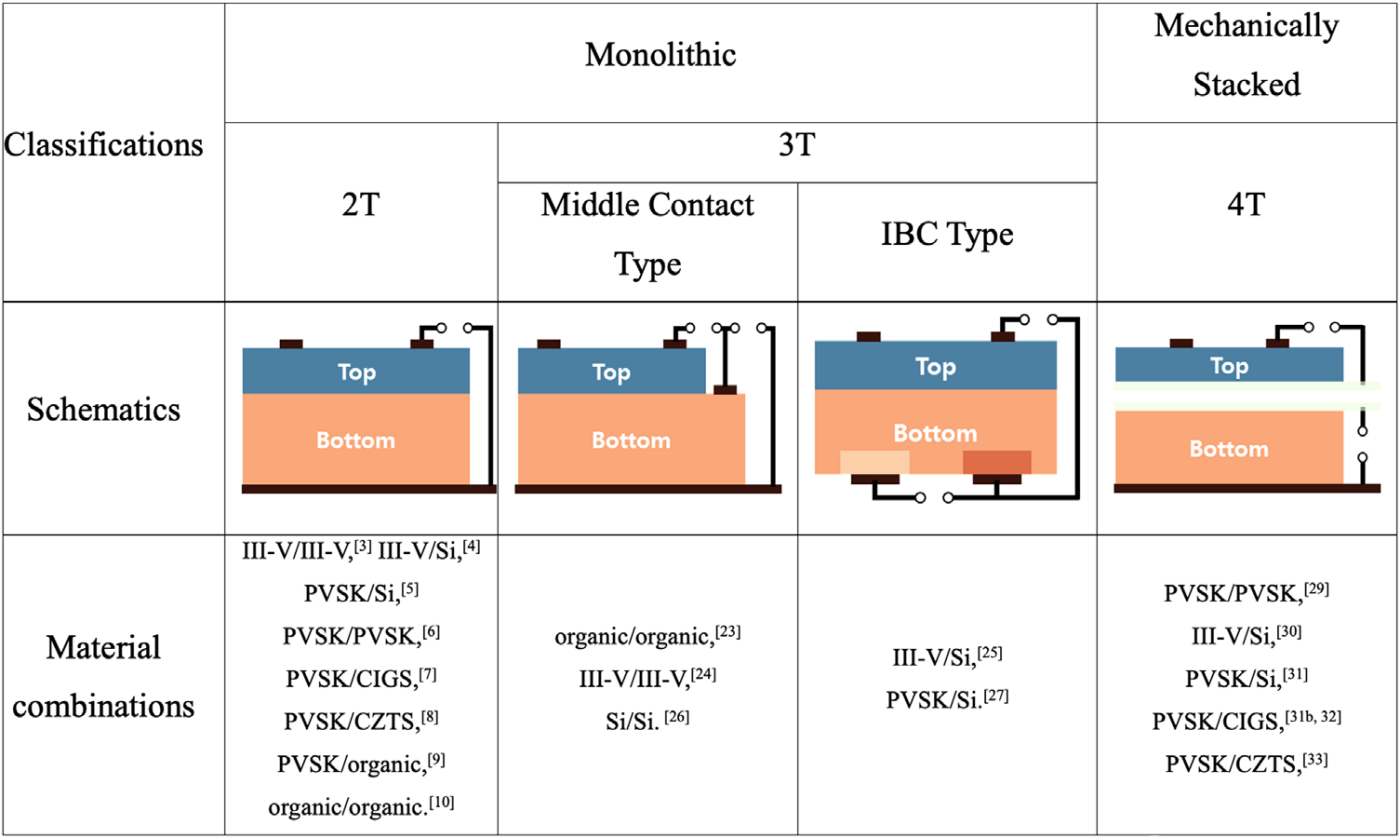
Classification of various tandem solar cells and their schematic structures.

TSCs can be classified as monolithic or mechanically stacked tandems based on the physical integration of the subcells. The subcells of a monolithic TSC are electrically connected, whereas each subcell of a mechanically stacked TSC functions independently. Monolithic TSCs often suffer from increased fabrication costs and technical difficulties for subcell integration, while mechanically stacked TSCs suffer from increased material costs and optical losses due to the additional electrodes and substrates. In terms of characterization, it is more practical to classify the TSCs according to the number of electrical contacts that can be connected to the external circuit. As the number of electrical contacts in general TSCs ranges from two to four, they are often classified as 2T, three‐terminal (3T), or four‐terminal (4T) tandems. 2T and 3T TSCs are generally monolithic, while 4T TSCs are mechanically stacked. The typical classifications of TSCs and their electrical configurations are shown in Figure 1.

## 3 2T TSCs

The 2T TSC is the most studied TSC as it can directly replace SJSCs in the conventional photovoltaic modules owing to the same external electrical connection. Meanwhile, its fabrication is relatively difficult owing to the monolithic integration of the subcells. Moreover, the series connection of the subcells in a 2T TSC requires delicate photocurrent matching between the subcells. Various types of 2T TSCs are reported including III‐V multijunction solar cells,^[^ ^3^ ^]^ III‐V/Si,^[^ ^4^ ^]^ PVSK/Si,^[^ ^5^, ^6^, ^7^, ^8^, ^9^, ^10^ ^]^ PVSK/PVSK,^[^ ^11^, ^12^, ^13^ ^]^ PVSK/CuIn_x_Ga_(1‐x)_Se_2_(CIGS),^[^ ^14^, ^15^ ^]^ PVSK/Cu_2_ZnSn(S,Se)_4_(CZTS),^[^ ^16^ ^]^ PVSK/organic,^[^ ^17^, ^18^, ^19^ ^]^ and organic/organic.^[^ ^20^, ^21^ ^]^ In a 2T TSC, the subcells are electrically connected in series through an interconnection layer, and the device can be characterized as a single‐junction device with a high‐power output. In general, the standard measurement protocols for such classical SJSCs can be utilized for the characterization of 2T TSCs.^[^ ^2^ ^]^ However, several critical issues such as the spectral mismatch, difficult subcell characterization, and electrical/optical interaction between subcells, exist for the accurate characterization of 2T TSCs.

**FIGURE 2:**
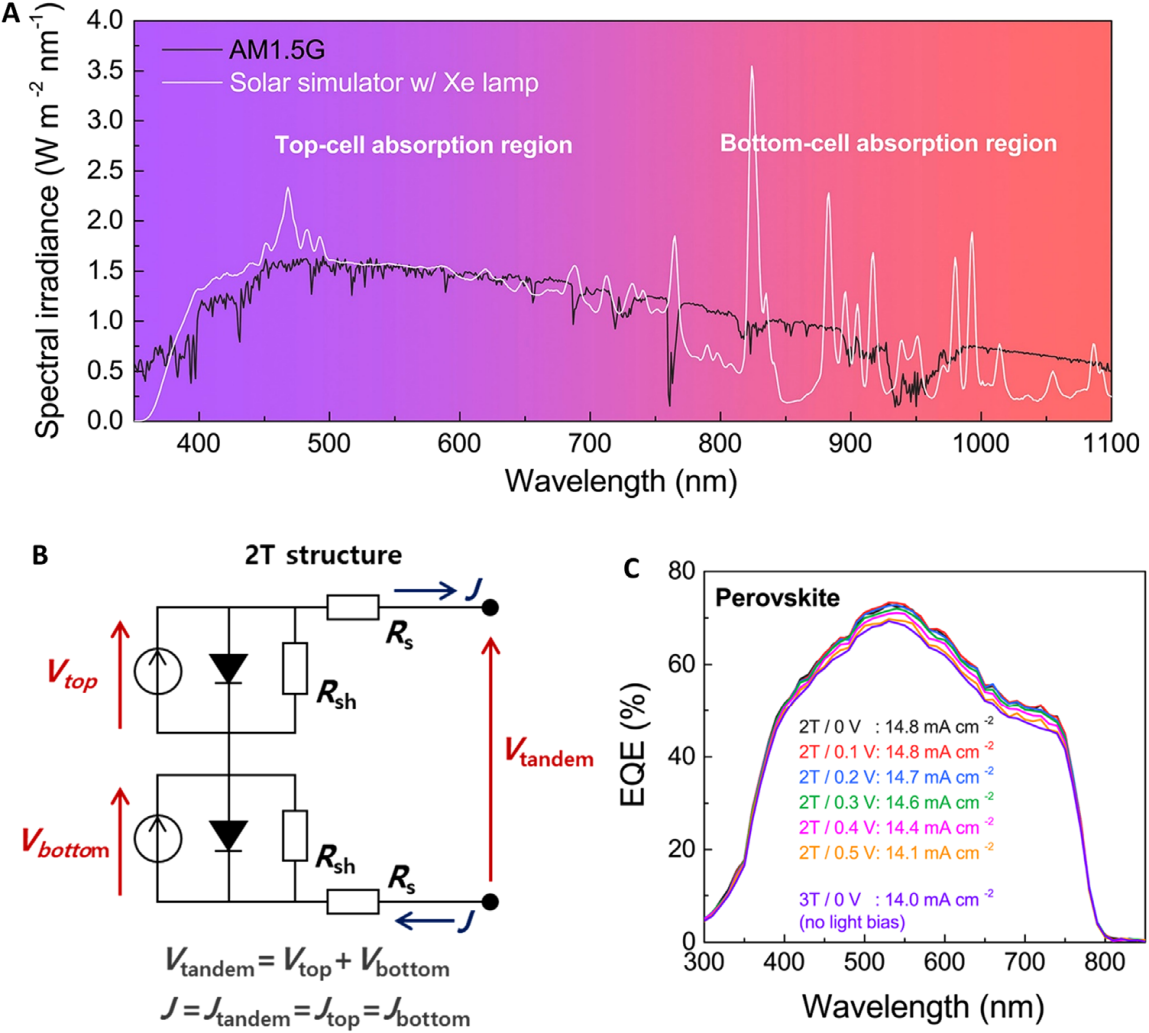
Characterization issues of 2T tandem solar cells (TSCs). (A) Spectral mismatch between spectral irradiance of the Sun (AM 1.5G) and a Xe‐lamp based solar simulator. Reproduced with permission.^[^ ^23^ ^]^ Copyright 2019, Elsevier Inc. (B) Equivalent circuit of the 2T TSC. (C) EQE spectra of PVSK top cell in a PVSK/Si 2T TSC depending on the electrical bias applied. Reproduced with permission.^[^ ^23^ ^]^ Copyright 2019, Elsevier Inc.

Most commercial solar simulators show a certain level of spectral mismatch compared with the standard solar spectra (e.g., ASTM G‐173),^[^ ^22^ ^]^ and should be adjusted to obtain accurate solar cell parameters. Typically, the incident light is adjusted by changing the light intensity with a certified reference cell to set the total number of photons entering the solar cell to the desired value. However, this strategy does not work for the TSCs because each subcell absorbs a respective range of incident light; changing the light intensity influences each subcell differently, resulting in a significant current mismatch (Figure 2A). Therefore, the only method to circumvent the spectral mismatch is to obtain the subcell current density from the external quantum efficiency (EQE) curve. However, independent subcell characterization of a 2T TSC is impossible owing to inadequate available electrical contact (Figure 2B). Instead, the subcell EQE curves must be measured by applying a light bias selectively to the subcell of no interest, thus, having the overall photoresponse limited by the subcell of interest. A carefully determined electrical bias should be simultaneously applied to the cell to compensate for the open‐circuit voltage (*V*_OC_) created by light bias (Figure 2C). The resulting short‐circuit current density (*J*_SC_) of the subcells can then be used to adjust the incident light by a light bias with appropriate wavelengths before measuring the solar cell performance of the 2T TSCs.

In addition to the conventional morphology/structure characterization methods such as scanning electron microscopy (SEM), transmission electron microscopy (TEM), and atomic force microscopy (AFM), which can be applied to the samples regardless of their electrical connection, and not specific to TSC, there are not many reported tools for the analysis of the 2T TSCs. To realize the characterization of the 2T TSC, one must be able to discern the responses of the subcells in the obtained results. One possible way is to analyze the luminescence of the device after electrical or optical excitation, that is, electroluminescence (EL) or photoluminescence (PL), respectively. As a semiconductor material emits photons of energy approximately equal to its bandgap when radiative recombination of carriers occurs, each subcell produces a distinct spectrum after its excitation. In the case of EL, an electrical current that is common to all series‐connected subcells is injected into the device to produce luminescence. Similarly, for PL, the subcells are excited by using a laser of the appropriate wavelength that is absorbed by only one subcell. These methods can provide the quasi‐Fermi level splitting (QFLS) of the subcells which can be interpreted as their *V*_OC_s.^[^ ^24^ ^]^ For PL, the QFLS is estimated based on the following expressions:

(1) $$\mathrm{QFL}S_{\mathrm{PL}}=\;k_{B}\;T\;ln\left(\mathrm{PLQY}\cdot \frac{\;J_{G}}{J_{0,\;\mathrm{rad}}}\right)$$

with *k*_B_ *T* being thermal voltage, PLQY being the photoluminescence quantum yield, *J*_G_ being the generation current density at the respective excitation intensity, and *J*_0,rad_ being the radiative thermal recombination current density estimated by integrating the overlap of the external quantum efficiency of the device (EQE) with the black body spectrum, that is,

(2) $$\begin{array}{c} \;J_{0,rad}=\int \mathrm{EQE}\phi_{\mathrm{BB}}\;d\in \;\mathrm{with}\;\phi_{\mathrm{BB}}=\frac{1}{4^{2}\hbar^{3}c^{2}}\cdot \frac{E^{2}}{\exp\left(\frac{E}{k_{B}T}\right)-1} \end{array}$$

For EL, the QFLS is estimated using a similar equation:

(3) $$\mathrm{QFL}S_{\mathrm{EL}}=\;k_{B}\;T\;ln\left(\mathrm{ELQ}E_{\mathrm{EL}}\cdot \frac{\;J_{\mathrm{inj}}}{J_{0,\;rad}}\right)$$

where ELQE_EL_ is electroluminescence quantum efficiency, and *J*_inj_ is injected current.

**FIGURE 3:**
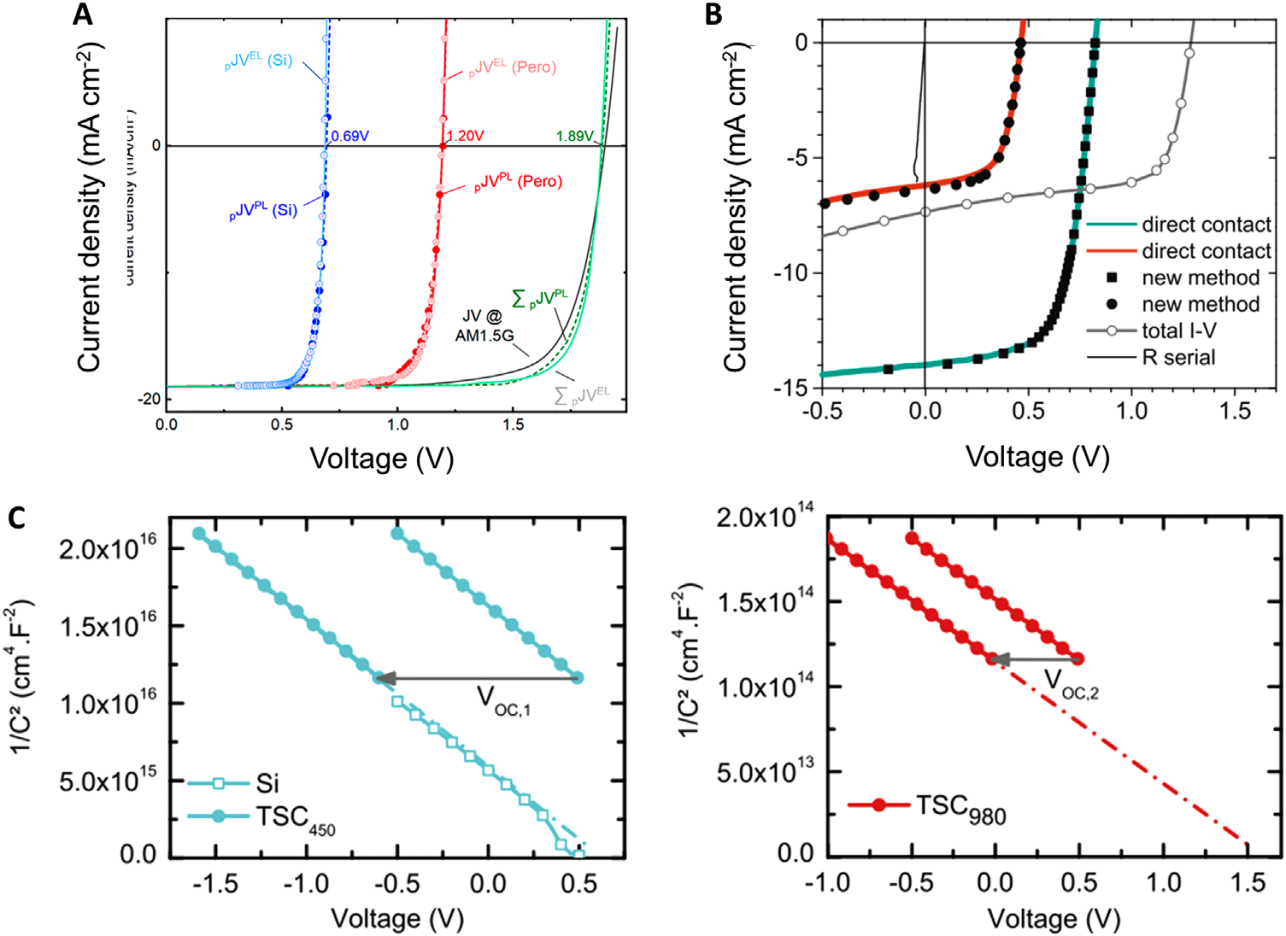
Subcell characterization of 2T tandem solar cells (TSCs). (A) Pseudo *J–V* characteristics of the subcells were obtained by EL and PL analysis (red‐perovskite, blue‐silicon), and experimentally measured *J–V* characteristics of the TSC (black line). Reproduced with permission.^[^ ^27^ ^]^ Copyright 2021, American Chemical Society. (B) Experimentally measured *J–V* of the TSC (black line) and *J–V* of the subcells (green and red lines) obtained by using additional light bias. Reproduced with permission.^[^ ^32^ ^]^ Copyright 2012, Elsevier B.V. (C) Mott‐Schottky plots of the subcells estimated by using additional light bias. Reproduced with permission.^[^ ^33^ ^]^ Copyright 2020, John Wiley & Sons, Ltd.

Further, the relationship between the QFLS and the light/current intensity can be analyzed to estimate the diode ideality factor of the subcells. The methods can also probe the photovoltaic properties of the subcell; the ideal *J–V* curves, also called pseudo‐ *J–V* curves of the subcells can be obtained through the analysis of the PL and EL,^[^ ^25^, ^26^ ^]^ which can provide insights on the characteristics of the subcells (Figure 3A). In these methods, the currents of the subcells are estimated by the following expressions:

(4) $$\;J_{\mathrm{PL}}=\frac{qP_{\mathrm{ex}}\mathrm{EQE}\left(E_{\mathrm{ex}}\right)}{E_{\mathrm{ex}}A_{\mathrm{ex}}}$$

(5) $$\;J_{\mathrm{EL}}=J_{\mathrm{inj}}$$

where *P*_ex_, *E*_ex_, and *A*_ex_ denote excitation laser power, energy per photon, and area of the excitation spot, respectively. The obtained currents and voltages, that is, QFLS are then combined with the *J*_SC_ value at 1 Sun resulting in the pseudo‐*J–V* curves.

For emerging TSCs, a similar study is reported by Al‐Ashouri et al.^[^ ^9^ ^]^ for PVSK/Si TSC wherein the hole transport of the top cell was largely improved and identified. Lang et al. extended the analysis by studying the efficiency limit of the subcells through the PL study performed on the samples with and without charge transport layers.^[^ ^27^ ^]^ Indeed, contrary to the EL which requires a complete device for effective charge injection, the PL can be obtained on incomplete devices to probe the diverse aspects of the samples such as *V*_OC_ limit and loss mechanism. Moreover, the PL measurement can be performed with a spatial resolution to identify the defective parts of the cell as well as the effect of its texture.^[^ ^28^, ^29^ ^]^

Furthermore, the electrical characterization of the subcell can be realized by using subcell‐selective light sources. When a device is under the light bias that is absorbed by only one subcell, the current of the device is largely limited by that of the unbiased subcell as the same amount of the current must flow through the device. Therefore, by applying a light bias of appropriate wavelength, the short‐circuit current density (*J*_SC_) of the current‐limiting subcell can be obtained through the *J–V* measurement of the device, as well as its open‐circuit voltage (*V*_OC_), which can result in the *J*_SC_–*V*_OC_ or Suns‐*V*_OC_, that is, pseudo‐*J–V* characteristics of the subcells (Figure 3B).^[^ ^30^, ^31^ ^]^ Based on this principle, Holovský et al. suggested a method that can estimate the *J–V* characteristics of the subcells of amorphous Si/microcrystalline Si TSC.^[^ ^30^, ^31^, ^32^ ^]^ They measured the voltage of the TSC under a constant light bias (1 Sun) with additional subcell‐selective light biases of various intensities and the injected current equal to the photocurrent created by the additional light bias. Moreover, Leon et al. showed that the capacitance‐voltage technique can be successfully applied to an AlGaAs/Si TSC to probe the doping densities of its subcells by applying a subcell‐selective light bias and measuring the capacitance of the TSC (Figure 3C).^[^ ^33^ ^]^ On the other hand, for the emerging 2T TSCs which often have poor diode characteristics compared to the crystalline III‐V or Si TSCs, only a few attempts of this approach are reported: Jošt et al.^[^ ^34^ ^]^ reported the use of a bichromatic light source to probe the diode characteristics of the subcells of PVSK/Si TSC. They measured the *J–V* of the TSC under various illumination conditions composed of two distinct LEDs (450 and 940 nm) to estimate the diode ideality factor, dark current, and shunt resistance of the subcells to finally reconstruct their *J–V* using diode equation.

Another simple but powerful strategy to measure most of the subcell properties, including *J–V*, EQE, and electron dynamics, is to use the internal recombination layer of the 2T TSC as an auxiliary contact for subcell characterization.^[^ ^23^ ^]^ Although the electrical configuration of this measurement platform is analogous to the series‐connected 3T TSC, the device is still a 2T TSC as the auxiliary contact is used only for the subcell characterization. All methods for the electrical analysis of the solar cells apply to subcell characterization using this pseudo‐3T characterization platform. Based on the study using a 3T PVSK/Si TSC and subcell‐selective light biases, Park et al. reported diverse characterization methods including *J–V* characteristics, impedance spectroscopy, and thermal admittance spectroscopy that can be applied to 2T PVSK/Si TSC.^[^ ^35^ ^]^ Meanwhile, as will be discussed below, luminescence and electric coupling must be considered.

## 4 **3T TSC**s

**FIGURE 4:**
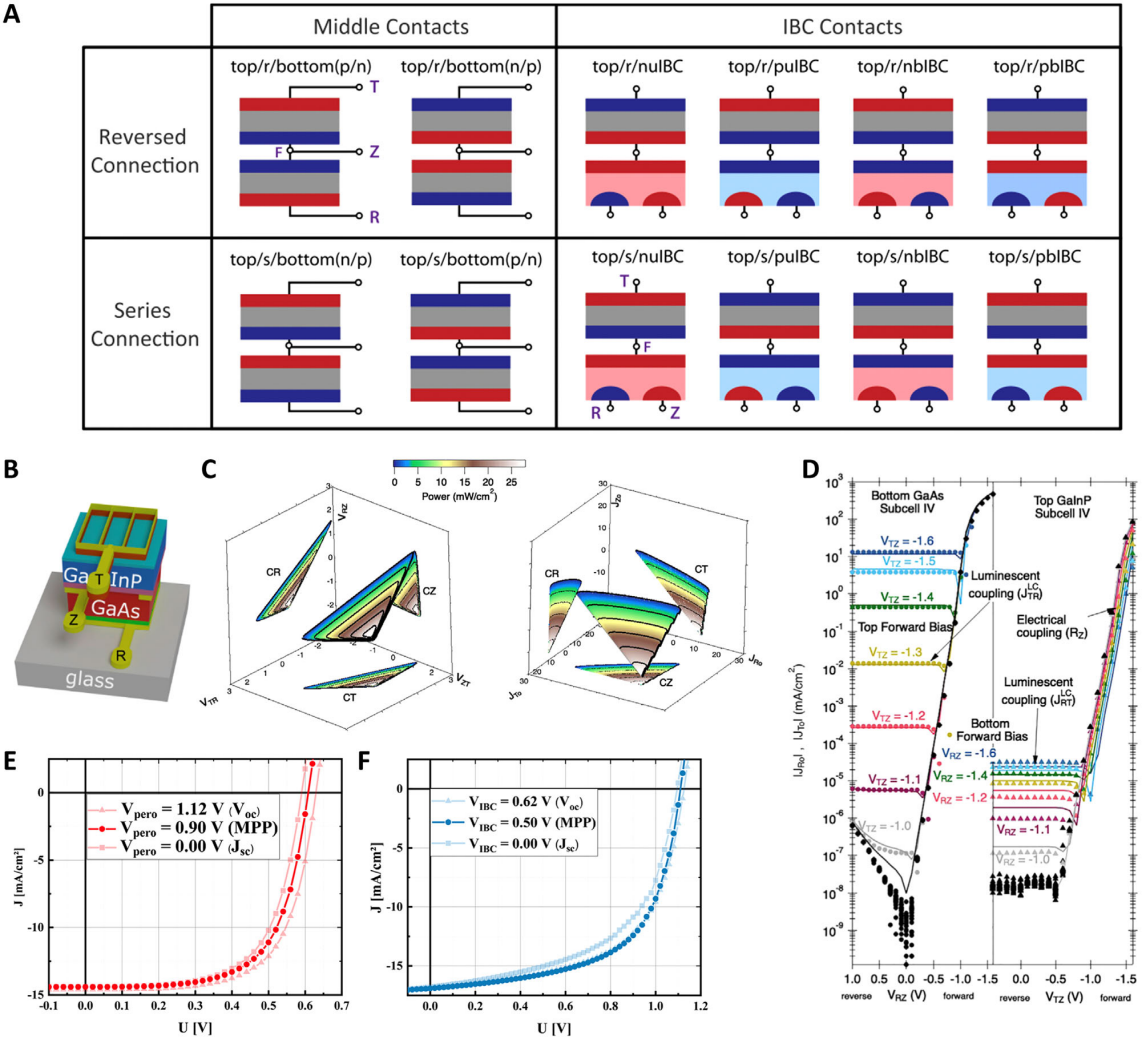
Characterization of 3T tandem solar cells (TSCs). (A) Mapping the wide variety of 3T TSC configurations. In all schematics, n‐type materials are red and p‐type materials as blue. “Top” is used as a representative of top cell, and in a real device would be replaced by the name of the material, for example, “perovskite” or “GaInP.” The purple letters (T, F, R, and Z) correspond to the names of the nodes used for different loading configurations. Reproduced with permission.^[^ ^36^ ^]^ Copyright 2020, American Chemical Society. (B) Three‐dimensional schematic showing external top (T), middle (Z), and rear (R) contacts. (C) Left: *P*_tot_(*V*_ZT_,*V*_RZ_,*V*_TR_), where voltages have units of volts. Right: *P*_tot_(*J*_Ro_, *J*_Zo_, *J*_To_), where current densities have units of milliamperes per square centimeter. The power density is indicated by the color bar, with black iso‐power contours every 5mWcm^−2^. Each raw dataset of (C) is plotted on its plane of origin (CZ, CR, or CT). The CZ measurements are projected onto the 3D device plane. (D) Dark *J–V* of the subcells as functions of the voltage on the other subcell, highlighting luminescent and electrical coupling. Reproduced under the terms of the CC‐BY‐NC‐ND 4.0 license.^[^ ^39^ ^]^ Copyright 2021, Elsevier. *J–V* characteristics of (E) IBC silicon subcell and (F) perovskite subcell while the respective other subcell is set to a certain bias. Reproduced with permission.^[^ ^42^ ^]^ Copyright 2020, American Chemistry Society.

Unlike the 2T TSC in which the subcells are connected in series, a 3T TSC design can be adopted in various ways owing to an additional electrode and source measure unit (SMU), enabling the collection of two individual power outputs (Figure 4A).^[^ ^36^ ^]^ However, these additional features require a new module design, potentially leading to a higher cost for commercialization. Until now, only a limited number of realized devices are reported as the concept is relatively new: organic/organic,^[^ ^37^ ^]^ III‐V/III‐V,^[^ ^38^, ^39^ ^]^ III‐V/Si,^[^ ^40^ ^]^ Si/Si,^[^ ^41^ ^]^ and PVSK/Si.^[^ ^42^ ^]^ Depending on the bottom cells, 3T TSCs can be grouped into either the interdigitated back contact (IBC) type, or the middle‐contact‐type. A typical monolithic IBC TSC features two electrical contacts at the rear side of the TSC and one contact at its front side, as shown in Figure 4A. Constructing 3T TSCs is the only feasible method to form TSCs with highly efficient IBC bottom cells where no top contact is available. Conversely, the middle‐contact‐type 3T TSC has an extra electrical contact positioned between the two subcells to either facilitate the characterization or for the parallel connection of the subcells, for which the active area of one subcell is reduced to expose the internal contact. The subcells can be either connected in series (i.e., tunnel junction type) or parallel (i.e., bipolar junction transistor type). Regardless of the configuration, two subcells of the 3T TSCs function separately using their own source measure units. Thus, a single 3T TSC produces two small power outputs rather than one high‐power output. Consequently, 3T TSCs are not strictly restricted by the current matching between subcells, unlike 2T TSCs. The sum of the two subcell power outputs is likely to exceed the power output of the 2T TSC if the subcell currents do not match well.^[^ ^43^ ^]^

Warren et al. suggested a taxonomy for the 3T TSCs by denoting the three electrical terminals as T, Z, and R depending on their positions and roles: T for top, R for rear, and Z for extra or middle.^[^ ^36^ ^]^ Moreover, considering that an electrode must be shared by the two SMUs used, they suggested to denote the possible measurement configurations as CT, CR, and CZ depending on the name of the common electrode chosen for example, CT for common top electrode. They also showed that the same power output is obtained regardless of the electrical connection used for the measurement by comparing the results from different configurations using high‐quality III‐V 3T TSCs (Figure 4B,C).^[^ ^39^ ^]^ Although the performance measurements were barely affected, electrical and optical coupling issues were observed (Figure 4D).

While only a limited number of 3T TSCs have been experimentally demonstrated,^[^ ^40^, ^42^ ^]^ there are some factors, such as luminescent and electrical coupling, that must be considered when measuring the 3T TSCs. In contrast to the 2T TSC, where the coupling between the subcells is naturally accounted for during the performance measurement, the subcells of the 3T TSC can exhibit coupled behaviors. For instance, different levels of radiative recombination can induce the top cell to emit different amounts of photons based on the applied voltage. Further, the luminescence from the top cell with wider bandgap energy can significantly influence the performance (e.g., the photocurrent) of the bottom cell with smaller bandgap energy, specifically when the radiative recombination is dominant in the top cell, as in the case of the III‐V top cells. However, the radiation from the bottom cell barely influences the top cell because the photon energy is smaller than the bandgap energy of the top cell. In addition to the luminescent coupling, another coupling issue may exist in 3T TSCs where one electrode is shared by two simultaneously operating SMUs. As can be seen from the case of a perovskite/IBC Si 3T TSC,^[^ ^42^ ^]^ the *V*_OC_, or fill factor, of a subcell can be influenced by the electrical bias applied to another subcell, presumably because of the changes in the charge transfer near the interface between the subcells (Figure 4E,F). Because the imbalance between electron and hole transfer rates strongly influences performance and stability, such electrical coupling problems should be considered more seriously for perovskite‐based TSCs. Therefore, to minimize the coupling issues between subcells, the performance of 3T TSCs must be measured when both cells are in the operation condition.

In middle contact type 3T TSCs, obtaining the subcell EQE curves, which are essential for adjusting the spectrum of the incident light is quite straightforward, and highly reliable results can be obtained without any electrical and light bias.^[^ ^23^ ^]^ However, the EQE measurement is more complicated for the IBC‐based 3T TSC owing to the absence of the top contact in the bottom cell. The EQE of the top cell must be influenced by the bottom cell because the top cell cannot be connected to the SMU independently as in 2T TSCs. On the other hand, the spectrum adjustment is not critically important for the 3T TSCs as they are much less sensitive to the current mismatch compared with the 2T TSC. Meanwhile, only a limited number of 3T TSCs are experimentally realized, especially with emerging solar cells and IBC‐type bottom cells. Thus, more realization of devices and their characterizations is necessary.

## 5 **4T TSC**s

**FIGURE 5:**
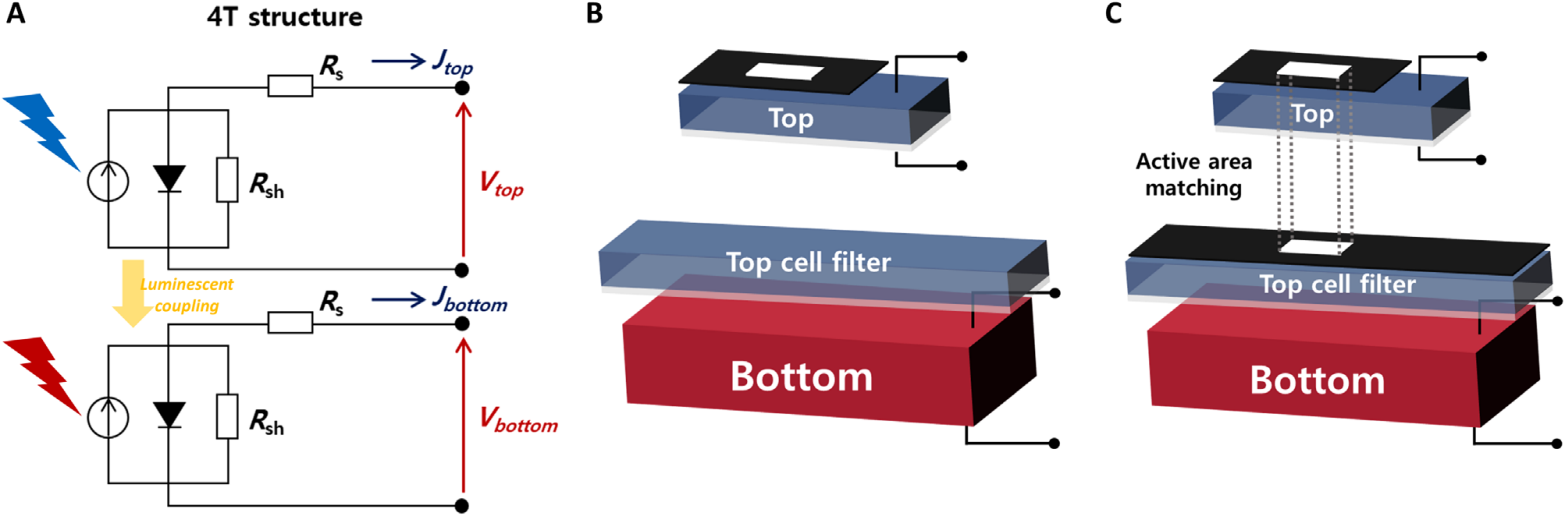
Characterization issues of 4T tandem solar cells (TSCs). (A) Equivalent circuit of the 4T TSC. Three‐dimensional schematics showing 4T TSC measurement scheme (B) with and (C) without matching the active area.

Two stacked solar cells can result in a 4T TSC provided that the rear side of the top cell is transparent since the interconnection between the subcells is not necessary for 4T TSC. Although the additional transparent electrode and the insulation between the subcells cause an optical loss and an increased module cost, the facile integrability is beneficial especially for the solar cells with limitations in their fabrication process. Similar to 3T TSCs, 4T TSCs require new module designs, such as a module consisting of two independent top (transparent) and bottom modules. Therefore, extremely large variety of 4T TSCs are reported: PVSK/PVSK,^[^ ^44^ ^]^ III‐V/Si,^[^ ^45^, ^46^ ^]^ PVSK/Si,^[^ ^47^, ^48^ ^]^ PVSK/CIGS,^[^ ^48^, ^49^ ^]^ PVSK/CZTS,^[^ ^50^ ^]^ etc. In 4T TSCs, two electrical contacts of each subcell (i.e., four contacts overall) can be freely connected to become equivalent to any of the 2T or 3T configurations. For example, they can operate either using one SMU or using two SMUs, and subcells can be connected either in series or in parallel. Therefore, according to the electrical connection, most of the advantages and limitations of 2T and 3T TSCs are also valid for 4T TSCs. Although characterization of 4T TSCs is much easier than that of other types of TSCs (Figure 5A), there are a few typical mistakes frequently observed in the literature. First, the subcells are sometimes measured separately. Similar to 3T TSCs, there is inevitable electrical and/or luminescent coupling between the subcells; thus, two subcells should be measured simultaneously to consider the coupling effect. To mimic actual operation conditions, when measuring one subcell, another subcell should be simultaneously illuminated by the incident light and applied with the electrical bias corresponding to its maximum power point voltage.^[^ ^46^, ^51^ ^]^ Second, the cell area may not be properly defined. Because the overall efficiency of 4T TSCs is obtained by adding the efficiencies of two separate subcells stacked together, the actual cell areas of the subcells are often significantly different from each other (Figure 5B,C). The efficiency of a solar cell is strongly influenced by cell area. Therefore, when reporting the conversion efficiency of a single 4T TSC, the efficiencies of the subcells should be measured with the same cell area.^[^ ^52^ ^]^

Meanwhile, as the subcells can operate independently in a 4T TSC, any electrical characterization can be applied to probe their characteristics which facilitates the optimization of the device performance. Moreover, similar to the 3T TSC, the 4T TSC shows satisfactory robustness against the spectrum variation of the incident light as both subcells can operate at their maximum power pointsadapting to the light condition when two SMUs are used.

## 6 CONCLUSION

In summary, interest in emerging TSCs is continuously increasing with their skyrocketing conversion efficiency leading to various types of TSCs being reported. However, because of their structural/electrical complexity and the absence of standard protocols, the accurate characterization of TSCs remains a challenge. Typical types of TSCs are classified as 2T, 3T, or 4T TSCs, based on the number of available electrical contacts that can be connected to the external circuit. For 2T TSCs, even though there are some applicable measurement standards, the spectral mismatch, coupling between subcells, and subcell characterization issues should be carefully considered during performance measurement. Further, the conversion efficiencies of 3T and 4T TSCs reported in the literature are the simple sum of efficiencies obtained from two subcells with separate SMUs, suggesting that two small power outputs rather than a single high‐power output are produced. In addition to the common issues, because of the various material combinations and electrical connections between them, 3T TSCs, in particular, require more studies to facilitate the standardization of measurement.

## CONFLICT OF INTEREST STATEMENT

The authors declare no conflicts of interest.
